# Bacterial profile and antibiotic susceptibility patterns in patients with secondary peritonitis: a cross-sectional study in Uganda

**DOI:** 10.1186/s13741-024-00425-4

**Published:** 2024-06-24

**Authors:** Nyenke Bassara Godefroy, Joshua Muhumuza, Selamo Fabrice Molen, Musa Abbas Waziri, ByaMungu Pahari Kagenderezo, Bienfait Mumbere Vahwere, Frank Katembo Sikakulya, William Mauricio, Joel Wandabwa, Bisingurege Kagoro Francois, Ezera Agwu, Xaviour Francis Okedi

**Affiliations:** 1 Department of Surgery, Faculty of Clinical Medicine and Dentistry, Kampala International University Western Campus, Ishaka-Bushenyi, Uganda; 2 Department of Surgery, Hoima Regional Referral Hospital, Hoima, Uganda; 3 Department of Internal Medicine, Faculty of Clinical Medicine and Dentistry, Kampala International University Western Campus, Ishaka-Bushenyi, Uganda; 4 Department of Microbiology, Faculty of Clinical Medicine and Dentistry, Kampala International University Western Campus, Ishaka-Bushenyi, Uganda; 5 Department of General Surgery, State Specialist Hospital, Borno State, Shehu Laminu Way, P.M.B, Maiduguri, 1014 Nigeria

**Keywords:** Secondary peritonitis, Risk factors, Bacteria isolates, Susceptibility

## Abstract

**Introduction:**

Secondary peritonitis is the second leading cause of sepsis worldwide. Drug resistance to peritoneal cavity bacterial infection remains a public health threat, especially in resource-limited settings in Africa, including Uganda. This study aimed to determine the antibacterial susceptibility patterns and factors associated with secondary peritonitis among patients with acute abdomen who underwent surgery at a Regional Referral Hospital in Uganda.

**Methods:**

This was a cross-sectional study conducted at Hoima Regional Referral Hospital (HRRH) that enrolled 126 patients with acute abdomen. Clinical samples were aseptically collected at laparotomy from patients with secondary peritonitis for culture and sensitivity using standard Microbiological methods. Binary logistic regression was used to identify factors associated with secondary peritonitis among patients with acute abdomen.

**Results:**

The majority of the patients were males (61.9%) with a mean age of 37.9(SD ± 21.8). Secondary peritonitis was found in 57(45.2%) of the patients. Gram-negative bacteria were the most commonly isolated organisms with *Escherichia coli* (35.8%) and *Klebsiella* spp (17.0%) predominating*.* Imipenem 88.8%(8/9), Amikacin 88.8%(8/9), Ciprofloxacin 44.4%(4/9) and Gentamicin 44.4%(4/9) demonstrated sensitivity to the different isolated organisms at varying degrees. Being a male (AOR = 3.658; 95% CI = 1.570–8.519, *p* = 0.003) and presenting 3 days after onset of symptoms (AOR = 2.957; 95% CI = 1.232–7.099, *p* = 0.015) were independently associated with secondary peritonitis.

**Conclusion:**

Imipenem, Amikacin, Ciprofloxacin, and Gentamicin should be considered for empirical therapy in cases of secondary peritonitis. Patients, more especially males with abdominal pain should be encouraged to present early to the hospital to minimize progression to secondary peritonitis.

**Supplementary Information:**

The online version contains supplementary material available at 10.1186/s13741-024-00425-4.

## Background

Acute abdomen refers to abdominal pain that starts abruptly typically lasting not more than 24 h requiring urgent attention (Kimuli 2011). Most of the causes of acute abdomen require surgical attention (Mabewa et al. 2015). Peritonitis is the inflammation of the peritoneum, the layer that encloses many organs in the abdomen (Tochie et al. 2020). There are three types of peritonitis: primary (spontaneous), secondary, and tertiary (Clements et al. 2021). Secondary peritonitis refers to irritation of the abdominal peritoneal lining caused by direct contact with peritoneal contaminants from gastrointestinal, intra-abdominal inflammation, or genitourinary system perforation (Mischianu et al. 2008). Secondary peritonitis is associated with complications such as surgical site infection, bust abdomen, re-laparotomy, prolonged hospitalization, morbidity, and mortality (Mabewa et al. 2015; Okidi et al. 2020).

For secondary peritonitis to occur, some factors come into play and these can be socio-demographic factors or medical factors (Emmanuel 2016; Appiah et al. 2020). Following secondary peritonitis, different bacterial organisms may be involved mainly depending on the cause of the peritonitis. Drug resistance to bacteria that cause infections of the peritoneal cavity is becoming a public health concern (Alelign et al. 2021). Although antimicrobial resistance containment interventions in healthcare structures have mostly been implemented in high-income countries, there is a pressing need to intervene in the antimicrobial resistance problem in low and middle-income countries (LMICs) such as Uganda (Sartelli et al. 2016).

Even though just 1% of all hospital admissions worldwide are due to secondary peritonitis, it is the most frequent reason for admission to the surgical wards, increasing workload, length of hospital stays, and consequences such as sepsis, surgical site infections, and enterocutanous fistula (Mabewa et al. 2015). Secondary peritonitis has also been reported to be the second leading cause of sepsis worldwide (Tochie et al. 2020; Alelign et al. 2021). In Africa, the proportion of secondary peritonitis has been reported to vary depending on the region with a prevalence of 19.1% in Ethiopia (Alelign et al. 2021), 23.7% in Tanzania (Seni et al. 2016), and 17.1% in Uganda (Amone et al. 2001).

Even though peritonitis has been extensively studied in developed countries (Rickard et al. 2018) there is still a paucity of data in low- and middle-income countries (LMICs) including Uganda and yet peritonitis is among the leading causes of mortality and morbidity among surgical patients (Muhumuza et al. 2023; Mutiibwa and Tumusiime 2013). Despite the known benefits of culture and sensitivity in the management of infections caused by secondary peritonitis, there is a paucity of data relating to bacterial isolates and sensitivity patterns, yet this information would be important in choosing empiric therapy. More so, an understanding of the factors associated with secondary peritonitis may help in reducing the number of patients that come in with secondary peritonitis. The primary objective of this study was to identify the microorganisms and their antibiotic susceptibility among patients with secondary peritonitis at Hoima Regional Referral Hospital in western Uganda. The secondary objective was to determine the antecedent determinants for secondary peritonitis among patients with acute abdomen who underwent surgery at Hoima Regional Referral Hospital in western Uganda.

## Materials and methods

### Study design

This was a cross-sectional study in which patients undergoing surgery for acute abdomen were enrolled. At laparotomy, in the patients in whom secondary peritonitis was diagnosed, a sample of peritoneal content was taken for culture and sensitivity. Also, patient data was taken to determine factors associated with secondary peritonitis.

### Study setting

This study was conducted at Hoima Regional Referral Hospital (HRRH), a public hospital funded by the Uganda Ministry of Health in which general care is free. It is located in Hoima Municipality, about 198 km west of Kampala with coordinates Latitude 1.428051 and Longitude 31.354451. The surgery department has one theatre room with an average of 800 surgeries per year. The department has 4 surgeons. One weekday is reserved for elective general surgeries but emergency surgeries are done whenever sanctioned. There are seven anesthetic officers. On average, 70 patients are operated for acute abdomen per month.

### Sample size determination and sampling

The sample size was calculated using Kish Leslie’s (1965) formula, $n=\frac{{Z}^{2}p(1-p)}{{d}^{2}}$ whereby, *n* = estimated minimum sample size required, *P* = proportion of characteristics in a sample (9%) according to a study in Northwestern Tanzania at University Teaching Hospital (Chalya et al. 2012), *Z* = 1.96 (for 95% Confidence interval), *d* = Margin of error set at 5%. On substituting, *n* = 126. Therefore the sample size required for this study was = 126. Convenience sampling was done in which consecutive patients who satisfied the eligibility criteria were enrolled till the required sample size of 126 was reached. The enrolment of participants lasted 4 months (July 2022 to October 2022).

### Inclusion and exclusion criteria

All patients who presented with the clinical and/or the radiological signs of acute abdomen that underwent surgery at Hoima Regional Referral Hospital were included if they consented. Patients with a history of abdominal trauma within 2 weeks before presentation and those who had been operated on within 1 month before presentation were excluded.

### Data collection procedure

A checklist was used to collect data from every patient who consented to participate. One part of the checklist collected information about demographic, socio-behavioral, medical, and environmental factors related to the development of secondary peritonitis. Another part collected information obtained from resuscitation, operation, swab collection, culture, and sensitivity results. The checklist was translated to Runyoro for the participants who could not understand English. The checklist was pre-tested for validity and reliability and necessary adjustments were made before data collection commenced.

Patients with acute abdomen requiring surgery were admitted to the emergency department of HRRH and informed about the study, written consent was sought, and detailed history was taken and documented in the checklist. In the supine position, physical examination for signs of secondary peritonitis, including guarding, rigidity, and tenderness on palpation of the abdomen, was done. The diagnosis was supported by radiological evidence and confirmed by intraoperative findings. The physiological status of the patient was assessed and resuscitation was done before transfer to the operating room. The procedure (exploratory laparotomy) was done under general anesthesia by a general surgeon assisted by a resident. A sterile swab stick was socked in the peritoneal fluid during laparotomy and immediately placed in the amies transport medium to ensure the possibility of capturing all the bacteria (Spencer et al. 2014). The research assistant transported the sample to the laboratory for immediate analysis after it had been labeled with the patient’s serial number. The swab was aseptically placed into the swab container with STUART media to maintain the viability of the aerobic and facultative anaerobic bacteria at the same time avoiding contamination (Alfa and Lee July 1981). The swab containers were placed in a cool box and transported to the microbiology laboratory of Kampala International University Teaching Hospital (KIU-TH) for laboratory analysis. The patient was given treatment according to Uganda’s clinical guidelines as the researcher continued to follow up on the result in the laboratory.

Samples collected using a sterile procedure with the peritoneal swab stick were inoculated on blood agar, chocolate agar, MacConkey agar, and Thayer Martin medium, and different biochemical tests were used. The culture media used was manufactured by HiMedia Labortories LLC 507 School House Rd., Suite 200, Kennett Square, PA 19348, USA. After that, they were incubated at 37 °C for 24–48 h both aerobically and anaerobically. The shape, size, height, margin, and surface properties of the colony were observed. The methods used to identify the organisms included gram staining, catalase test, indole test, coagulase test, citrate utilization test, urease test, triple sugar iron agar fermentation test, and oxidase test.

Pure culture colonies of the organisms were tested for their antibacterial drug susceptibility against 12 commonly used antibiotics using Kirby-Bauer disc diffusion as modified by the clinical laboratory standard institute. Selection of 3 to 5 isolated colonies from the medium was done using a sterile wire loop and transferred into 3 mls of nutrient broth in a bijou bottle. The broth was mixed by inversion for the complete dissolution of the colonies. The mixture was incubated for 4 min and was compared with 0.5% McFarland turbidity standard. A sterile inoculation glass rod was used to spread the surface of Muller-Hinton agar plates homogenously with the diluted colonies. Antibiotic discs were placed onto the inoculated Muller-Hinton agar and incubated at 37 ℃ for 24–48 h. The plates were examined and the diameter of zones of inhibition was measured in mm using a meter ruler and was compared to a standard chart of the corresponding antibiotics used for measuring zones of inhibition. The zone of inhibition was measured and recorded as susceptible (S), intermediate (I), or resistant (R) according to the standard chart. The Clinical and Laboratory Standards Institute (CLSI) guidelines were used to interpret the results of antibiotic susceptibility testing.

### Study variables

The independent variables included social demographic characteristics, medical characteristics, and the behavioral characteristics of the patients presenting with acute abdomen. The dependent variable was the occurrence of secondary peritonitis for which cases the bacterial isolates and susceptibility patterns were determined.

### Quality control and analysis

The questionnaire was pretested and necessary changes were made before starting data collection. The study assistants were trained before the study began. Data was checked for completeness at the end of each entry. Laboratory analysis was done by a qualified microbiologist with a master’s degree in microbiology. The culture media were checked for sterility and growth performance in addition to checking the pH value. The data was analyzed with the guidance of a biostatistician.

Data from the checklist were entered in Microsoft Excel 2010 and thereafter exported to SPSS version 22 for Windows. The proportion of secondary peritonitis among patients with acute abdomen who underwent surgery at Hoima Regional Referral Hospital was computed as a percentage of patients with secondary peritonitis of all patients with acute abdomen. The bacterial isolates were summarized as percentages, and frequencies, and presented in a table**.** The susceptibility pattern was summarized by various bacterial isolates and presented using a table. The factors associated with secondary peritonitis were analyzed by both bivariate and multivariate backward logistic regression analysis. Biologically plausible variables and those with *p* values < 0.2 were considered for multivariate analysis to avoid leaving out significant variables. The variables in the final multivariate model were significant when the *p* value was ≤ 0.05. The measure of association was reported as odds ratios (ORs) with corresponding 95% CI and *p* values. All statistical analyses were carried out in the SPSS 22 series for Windows.

### Ethical considerations and consent

All methods were carried out per relevant guidelines and regulations. Ethical approval was approved by the Research and Ethics Committee of Kampala International University Western Campus (Ref No: KIU-2022–121). Informed consent was obtained from all the participants and their legal guardians involved in the study.

## Results

This study enrolled 126 patients with acute abdomen who had a mean age of 37.9 (SD ± 21.8) years, with the majority being males (61.9%). Of the 126, only 57 were found to have secondary peritonitis accounting for 45.24% of the study participants. Peritonitis was evidenced by the presence of pus in the peritoneal cavity, the color/consistency of the peritoneal fluid, and evidence of organ/tissue damage such as a ruptured appendix or perforated bowel. The causes of secondary peritonitis were: gastric perforation in 19(33.3%), ileal perforation in 15(26.3%), Jejunal perforation in 5(8.8%), raptured appendix in 7(12.3%), appendicular abscess in 4(7.0%), gangrene due to intestinal obstruction in 5(8.8%), duodenal perforation in 1(1.8) and gall bladder gangrene in 1(1.8%). Of the 57 samples taken for culture and sensitivity, only 53 had growth and therefore, sensitivity patterns were assessed for the 53 participants. Gram-negative bacteria represented (66.7%) of isolated bacteria. Of the 53(93.0%) that had growth, *Escherichia coli* (*E. coli*) accounted for the majority 19(35.8%) followed by *Klebsiella* spp 9(17.0%), *Staphylococcus aureus* 7(13.2%), *Citrobacter* spp 5(9.4%), *Proteus* spp 4(7.5%), *Pseudomonas* spp 4(7.5%), *Enterobacter* spp 3(5.7%), *Enterococcus* spp 1(1.9%), and S*treptococcus* spp 1(1.9%).

### Antibacterial susceptibility patterns among patients with secondary peritonitis who underwent surgery at HRRH

In this study, all organisms isolated had complete resistance to cloxacillin, methicillin, ceftriaxone, amoxiclav, cefixime, penicillin, ampicillin, and metronidazole. The antibiotics that demonstrated effectiveness at varying degrees to the different organisms isolated were Gentamicin, Ciprofloxacin, Amikacin, and Imipenem. *Enterobacter* spp was only sensitive to Ciprofloxacin and Imipenem while S*treptococcus* spp was only sensitive to Imipenem and Amikacin. The details of susceptibility testing are shown in Table 1 below.

**Table 1:** Antibacterial susceptibility patterns among patients with secondary peritonitis who underwent surgery at HRRH

		***Pseudomonous* spp *N* = 4(7.5%)**	***Staphyococus aureus N*** = **7(13.2%)**	***Citrobacter* spp* N*** = **5(9.4%)**	***Enterococcus fecalis N*** = **1(1.9%)**	***E. coli N* = 19(35.8%)**	***Proteus N*** = **4(7.5%)**	***Enterobacter* spp *N*** = **3(5.7%)**	***Klebshiella* spp *N*** = **9(17.0%)**	***Streptococus* spp *N*** = **1(1.9%)**
**Imipenem**	S	25.0%	85.7%	0.0%	100%	94.7%	100%	66.7%	88.9%	100%
	I	0.0%	0.0%	0.0%	0.0%	0.0%	0.0%	0.0%	11.1%	0.0%
	R	75.0%	14.3%	100%	0.0%	5.3%	0.0%	33.3%	0.0%	0.0%
**Amikacin**	S	100%	14.3%	60.0%	100%	78.9%	50.0%	0.0%	55.6%	100%
	I	0.0%	0.0%	20.0%	0.0%	5.3%	0.0%	0.0%	0.0%	0.0%
	R	0.0%	85.7%	20.0%	0.0%	15.8%	50.0%	100%	44.4%	0.0%
**Ciprofloxacin**	S	0.0%	0.0%	20.0%	0.0%	5.3%	0.0%	33.3%	22.2%	0.0%
	I	0.0%	0.0%	0.0%	0.0%	5.2%	0.0%	0.0%	0.0%	0.0%
	R	100%	100%	80.0%	100%	89.5%	100%	66.7%	77.8%	100%
**Gentamicin**	S	50.0%	0.0%	0.0%	0.0%	31.6%	25.0%	0.0%	22.2%	0.0%
	I	0.0%	0.0%	0.0%	25%	0.0%	0.0%	0.0%	0.0%	0.0%
	R	50.0%	100%	100%	75%	68.4%	75.0%	100%	77.8%	100%
**Cloxacillin**	R	100%	100%	100%	100%	100%	100%	100%	100%	100%
**Methicillin**	R	100%	100%	100%	100%	100%	100%	100%	100%	100%
**Ceftriaxone**	R	100%	100%	100%	100%	100%	100%	100%	100%	100%
**Amoxiclav**	R	100%	100%	100%	100%	100%	100%	100%	100%	100%
**Cefixime**	R	100%	100%	100%	100%	100%	100%	100%	100%	100%
**Penicillin**	R	100%	100%	100%	100%	100%	100%	100%	100%	100%
**Ampicillin**	R	100%	100%	100%	100%	100%	100%	100%	100%	100%
**Metronidazole**	R	100%	100%	100%	100%	100%	100%	100%	100%	100%

*S* Sensitive,* I* Intermediate, *R* Resistant

### Factors associated with secondary peritonitis among patients with acute abdomen who underwent surgery at HRRH

At bivariate analysis, the variables that had a *p* value less than 0.2, and therefore qualified for multivariate analysis were sex, marital status, education level, chronic illness, time to presentation, use of traditional medication, physical exercise, type of house and the number of meals taken per day. The results of the bivariate analysis are shown in Supplementary file 1: Table S1a–c.

In multivariate analysis, the factors that were independently associated with the occurrence of secondary peritonitis were sex and time to presentation. According to our findings, a male patient with acute abdomen was 3.658 (CI = 1.570–8.519, *p* = 0.003) times more likely to have secondary peritonitis compared to a female patient. A patient who took 3 days or more to come to the hospital after the onset of symptoms was also found to be 2.957(CI = 1.232–7.099, *p* = 0.015) times more likely to have secondary peritonitis compared to one that presented in less than 3 days. The rest of the multivariate analysis is shown in Table 2.

**Table 2:** Multivariable analysis of variables associated with secondary peritonitis among patients with acute abdomen who underwent surgery at HRRH

Characteristic	Bivariate analysis			Multivariate analysis		
	cOR	80% CI	*P* value	AOR	95% CI	*P* value
**Sex**						
**Male**	**3.484**	**1.600–7.587**	**0.002**	**3.658**	**1.570–8.519**	**0.003**
Female	Ref					
**Marital status**						
Single	Ref					
Married	1.054	0.500**–**2.223	0.890	2.054	0.400**–**3.223	0.690
Widowed	0.136	0.016**–**1.172	0.069	0.236	0.026**–**2.172	0.569
Separated	0.362	0.035**–**3.735	0.394	0.462	0.015**–**4.735	0.294
**Education level**						
Never	Ref					
Primary	1.389	0.570**–**3.386	0.470	2.389	0.470**–**4.386	0.670
Secondary	2.083	0.788**–**5.506	0.139	3.083	0.688**–**6.506	0.339
University	N/A			N/A		
**Chronic illness**						
None	Ref					
PUD	2.227	0.993**–**4.994	0.052	3.227	0.793**–**5.994	0.252
HIV	1.485	0.282**–**7.810	0.641	2.485	0.182**–**8.810	0.741
Diabetes mellitus	N/A			N/A		
**Time to presentation (days)**						
< 3.0	Ref					
**3.1 + **	**3.288**	**1.510–7.159**	**0.003**	**2.957**	**1.232–7.099**	**0.015**
**Traditional medication**						
No	Ref					
Yes	3.368	1.602**–**7.084	0.001	1.698	0.714**–**4.037	0.231
**Physical exercise**						
No	Ref					
Yes	3.221	0.601**–**17.272	0.172	3.130	0.484**–**20.242	0.231
**House type**						
Permanent	Ref					
Semi-permanent	1.632	0.789**–**3.375	0.187	1.010	0.406**–**2.513	0.983
**Meals per day**						
< **3.0**	Ref					
3.1 +	0.605	0.285**–**1.281	0.189	0.741	0.308**–**1.783	0.504

*cOR*  Crude odds ratio, *AOR* Adjusted odds ratio, *CI* Confidence interval, *Ref*  Reference category, *N/A* Not applicable since the category did not register one of the outcomes

## Discussion

In this study, the proportion of patients with secondary peritonitis was high (45.2%). Compared to the proportion found in this study, Seni et al. (2016) in Tanzania found a higher proportion of secondary peritonitis (57.7%). In Ethiopia, two studies reported lower proportions of secondary peritonitis (19.3%) (Negash 2019) and 24% (Gebrie., et al. 2019). A recent study in Tanzania also reported a lower proportion (21.5%) (Kibunto et al. 2022). Contrary to our findings, Nyundo et al. (2013) in Rwanda reported a proportion of 41.5% related to poor knowledge of lower-level health professionals on the diagnosis and early decision for management of acute abdomen. The high proportion in our study could be explained by the late presentation related to some behaviors such as self-treatment, use of herbal treatment, and financial constraints typical of low-resource settings and low-income countries.

In this study, gram-negative bacteria represented (66.7%) of isolated bacteria which was comparable to the findings in a study conducted in Ethiopia by Alelign et al. (2021), where gram-negative organisms accounted for 76.6%. *Escherichia coli* (35.8%) and *Klebsiella* spp (17.0%) were identified as the commonest bacteria cultured from infected peritoneal fluid. These findings are similar to the study done in Ethiopia by Alelign et al. (2021) which reported *E. coli* (36.67%) and *Klebsiella* (20%) to be the commonest isolates. Furthermore, other studies conducted in Tanzania by Seni et al. (2016), in Nigeria by Akujobi et al. (2006), and in India by Kumar-m et al. (2021) confirmed the predominance of *E. coli* followed by *Klebsiella* spp as the most frequent bacteria growth from peritoneal infected fluid associated with secondary peritonitis. In contrast, a study done in Mbarara by Mutiibwa and Tumusiime, found the most common bacteria to be *Klebsiella* spp (37.9%) followed *by E. coli* (26.4%) in small bowel perforation as the cause of secondary peritonitis. The predominance of these bacteria species in the infected peritoneal fluid of patients with secondary peritonitis might be due to their presence as normal flora in the gastrointestinal tract.

All the nine bacteria isolated from infected peritoneal fluid due to secondary peritonitis had resistance to cloxacillin, methicillin, ceftriaxone, amoxiclav, cefixime, penicillin, ampicillin, and metronidazole. This implies that these commonly prescribed antibiotics cannot be used as first-line empirical therapy for secondary peritonitis, particularly in the study area. The practice of prescribing broad-spectrum antibiotics with no clear indication and over-the-counter use of antibiotics might have contributed to the resistance of these bacteria to these antibiotics which are normally readily available and affordable for the management of these strains of bacterial isolates. The antibiotics that demonstrated effectiveness at varying degrees to the different organisms isolated were Imipenem, Amikacin, Ciprofloxacin, and Gentamicin which is slightly close to the result found by Alelign in Ethiopia and Mutiibwa at Mbarara Regional Referral Hospital in Uganda (Alelign et al. 2021; Mutiibwa and Tumusiime 2013).

*E. coli* was highly sensitive to Imipenem (94.7%) and Amikacin (78.9%), with low sensitivity to Gentamicin (31.6%) and Ciprofloxacin (5.3%). This result is similar to a study done in India by Kumar-m et al. (2021) and Sheikhbahaei et al. (2014), in Iran where Imipenem (_ ≥ 95.6%), Amikacin (_ ≥ 95.6%), Gentamicin and Ciprofloxacin (≥ 60%) had high sensitivities to *E. coli*. In this study, the difference could be explained by the irrational prescription of Gentamicin and ciprofloxacin in our medical setting and also the fact that these drugs are cheaper and readily available than Imipenem and Amikacin.

*Klebsiella* spp were sensitive to Imipenem (100%) and amikacin (55.6%) but with slight sensitivity to Gentamicin (22.2%) and Ciprofloxacin (22.2%). Kumar-m et al. (2019) in India reported similar results as Dwihantoro and Rochadi (2016) in Indonesia but with some differences in antibiotic susceptibilities which included cephalosporin. These differences could be explained by the fact that the practices of antibacterial use have been shown to vary which can result in different patterns of resistance.

Literature shows that many factors are known to be associated with secondary peritonitis such as demographic, social behavior, medical, and even environmental factors (Emmanuel 2016; Appiah et al. 2020). In this study, two factors male gender and time to presentation were the significant risk factors associated with secondary peritonitis.

A male patient with acute abdomen was 3.658 (*CI* = 1.570–8.519, *p* = 0.003) times more likely to have secondary peritonitis compared to a female. This finding is similar to the research done in Uganda by Ojuka et al. (2014) in Nsambya Hospital where the male-to-female ratio was 3:1 for peritonitis. Other studies conducted in Tanzania by Mabewa et al. (2015) and Mukherjee and Sarkar (2016) in India found similar results of male-to-female ratios of 1.8:1 and 8.4:1.6 respectively. This could be explained by the poor health-seeking behavior of male patients (Mabewa et al. 2015; Mukherjee and Sarkar 2016) resulting in late presentation to the health facilities and the associated complications. Men are known to be more unworried than women about their health, which means they might spend more time with a medical condition before they decide to search for appropriate management. Research done by Fillingim et al. (1999) at Alabama University showed that women were more likely to worry about pain and feel more helpless about it, and are more likely than men to have depression and anxiety, all of which can lead to higher pain levels and health care seeking (Fillingim et al. 1999).

A patient who took 3 days or more to come to hospital after onset of symptoms was also found to be 2.957(CI = 1.232–7.099, *p* = 0.015) times more likely to have secondary peritonitis compared to the one who presented in less than 3 days which is in agreement with a study done in Mbarara Regional Referral Hospital (Mutiibwa and Tumusiime 2013). Other studies by Nansubuga et al. (2016) at Mulago National Referral Hospital, Mabewa et al. (2015) in Tanzania, and Ndayizeye et al. (2000) in Rwanda also had similar findings suggesting that late presentation could reflect delay in seeking health care, attempted treatment through a traditional healer, lack of resources for transport to the health facility and late referral by the peripheral health facilities. In some African settings, late presentation (beyond 24 h of the onset of the symptom) has been the norm, as seen at the study center.

## Conclusion

Secondary peritonitis is a common surgical emergency among patients with acute abdomen at Hoima Regional Referral Hospital and its management needs urgent surgical attention. *E. coli* and *Klebsiella* spp were the most common bacteria found in the infected peritoneal fluid of patients with secondary peritonitis after culture. These bacteria showed multiple resistances to the most commonly used antibiotics but were sensitive to Imipenem, Amikacin, Gentamicin, and Ciprofloxacin at varying degrees. Male sex and time to presentation to the hospital were the two main factors found to be independently associated with secondary peritonitis among patients having acute abdomen.

### Study limitations

This was a cross-sectional study, so the outcome related to antibiotics found effective after culture and sensitivity could not be determined. Depending on the culture media used, we were able to isolate aerobics and facultative anaerobes. Therefore, strict anaerobes and fungi were not captured which might explain why some samples did not yield any growth. Though we had high resistance levels, we did not subject the isolated organisms to any molecular tests to determine the possible presence of genes responsible for resistance, however, our findings can be used as a basis for another study to assess these.

### Recommendations

Imipenem, Amikacin, Ciprofloxacin, and Gentamicin should be considered for empirical therapy in cases of secondary peritonitis. Patients, more especially males with abdominal pain should be encouraged to present early to the hospital to minimize progression to secondary peritonitis. All health workers should participate in antibiotic stewardship to prevent the emergence, spread, and persistence of antibiotic resistance. We recommend that a country-wide (multi-center) study be done on antimicrobial resistance and antibiotic susceptibility in select regional hospitals, both public and private to guide policy shifts on prescription in addition to determining the resistant genes of the isolates. There should be periodic monitoring of antimicrobial resistance patterns to help physicians choose antimicrobial agents for empiric treatment of secondary peritonitis.

### Supplementary Information

Supplementary Material 1.

## Data Availability

Data is available upon request. Requests should be sent to NBG Via nyenkegodefroy@yahoo.fr.

## Abbreviations

HRRH: Hoima Regional Referral Hospital
LMIC: Low- and middle-income countries
