# Supplementary material for: Bacterial profile and antibiotic susceptibility patterns in patients with secondary peritonitis: a cross-sectional study in Uganda
Source: Perioper Med (Lond). 2024 Jun 24;13:62. doi: 10.1186/s13741-024-00425-4 (PMC11197276; doi:10.1186/s13741-024-00425-4)
Supplement: Supplementary file 1 — Supplementary Material 1. [file 13741_2024_425_MOESM1_ESM.docx]

**Table S1a: Bivariate analysis of social demographic variables associated with secondary peritonitis among patients with acute abdomen who underwent surgery at HRRH**

| Variables | No Secondary peritonitis  N=69  n (%) | Secondary peritonitis, N=57  n (%) | Bivariate analysis | | |
| --- | --- | --- | --- | --- | --- |
|  |  |  | **cOR** | **80% CI** | **P value** |
| Age Category | |  |  |  |  |
| 0-16 | 12(50.0) | 12(50.0) | Ref |  |  |
| 17-30 | 17(58.6) | 12(41.4) | 0.706 | 0.238-2.098 | 0.531 |
| 31-45 | 15(53.6) | 13(46.4) | 0.867 | 0.291-2.582 | 0.797 |
| 46 and above | 25(55.6) | 20(44.4) | 0.800 | 0.296-2.159 | 0.660 |
| Sex |  |  |  |  |  |
| Male | **34(43.6%)** | **44(56.4%)** | **3.484** | **1.600-7.587** | **0.002** |
| Female | 35(72.9%) | 13(27.1%) | Ref |  |  |
| Marital Status |  |  |  |  |  |
| Single | 25(52.1%) | 23(47.9%) | Ref |  |  |
| Married | 33(50.8%) | 32(49.2%) | 1.054 | 0.500-2.223 | 0.890 |
| Widowed | **8(88.9%)** | **1(11.1%)** | **0.136** | **0.016-1.172** | **0.069** |
| Separated | 3(75.0%) | 1(25.0%) | 0.362 | 0.035-3.735 | 0.394 |
| Religion |  |  |  |  |  |
| Protestant | 18(47.4%) | 20(52.5%) | Ref |  |  |
| Catholic | 34(58.6%) | 24(41.4%) | 2.222 | 0.185-26.629 | 0.529 |
| Born again | 10(55.6%) | 8(44.4%) | 1.412 | 0.121-16.470 | 0.783 |
| Muslim | 5(55.6%) | 4(44.4%) | 1.600 | 0.122-20.993 | 0.720 |
| Others | 2(66.7%) | 1(33.3%) | 1.600 | 0.104-24.703 | 0.736 |
| Education level |  |  |  |  |  |
| Never | 20(62.5%) | 12(37.5%) | Ref |  |  |
| Primary | 30(54.5%) | 25(45.5%) | 1.389 | 0.570-3.386 | 0.470 |
| Secondary | **16(44.4%)** | **20(55.6%)** | **2.083** | **0.788-5.506** | **0.139** |
| University | 3(100%) | 0(0.0%) | N/A |  |  |
| Occupation |  |  |  |  |  |
| peasant | 37(52.9%) | 33(47.1%) | Ref |  |  |
| Formal employment | 15(68.2%) | 7(31.8%) | 0.523 | 0.190-1.440 | 0.210 |
| Student | 15(55.6%) | 12(44.4%) | 0.897 | 0.367-2.190 | 0.811 |
| Business | 2(28.6%) | 5(71.4%) | 2.803 | 0.509-15.431 | 0.236 |

**Table S1b: Bivariate analysis of medical variables associated with secondary peritonitis among patients with acute abdomen who underwent surgery at HRRH**

| Variables | No Secondary peritonitis  N=69  n (%) | Secondary peritonitis, N=57  n (%) | Bivariate analysis | | |
| --- | --- | --- | --- | --- | --- |
|  |  |  | **cOR** | **80% CI** | **P value** |
| Chronic illness |  |  |  |  |  |
| None | 49(59.8%) | 33(40.2%) | Ref |  |  |
| PUD | **14(40.0%)** | **21(60.0%)** | **2.227** | **0.993-4.994** | **0.052** |
| HIV | 3(50%) | 3(50.0%) | 1.485 | 0.282-7.810 | 0.641 |
| Diabetes Mellitus | 3(100%) | 0(0.0%) | N/A |  |  |
| Chronic medication | |  |  |  |  |
| None | 63(53.8%) | 54(46.2%) | Ref |  |  |
| ART | 3(50.0%) | 3(50.0%) | 1.167 | 0.226-6.021 | 0.854 |
| Hypoglycemic | 3(100%) | 0(0.0%) | N/A |  |  |

**Table S1c: Bivariate analysis of behavioral and environmental variables associated with secondary peritonitis among patients with acute abdomen who underwent surgery at HRRH**

| Variables | No Secondary peritonitis  N=69  n (%) | Secondary peritonitis, N=57  n (%) | Bivariate analysis | | |
| --- | --- | --- | --- | --- | --- |
|  |  |  | **cOR** | **80% CI** | **P value** |
| Time to presentation (days) | |  |  |  |  |
| < 3.0 | 34(72.3%) | 13(27.7%) | Ref |  |  |
| 3.1+ | **35(44.3%)** | **44(55.7%)** | **3.288** | **1.510-7.159** | **0.003** |
| Traditional medication use | | |  |  |  |
| No | 50(66.7%) | 25(33.3%) | Ref |  |  |
| Yes | **19(37.3%)** | **32(62.7%)** | **3.368** | **1.602-7.084** | **0.001** |
| Consult a friend/relative before coming to the hospital | | |  |  |  |
| No | 27(55.1%) | 22(44.9%) | Ref |  |  |
| Yes | 42(54.5%) | 35(45.5%) | 1.023 | 0.498-2.101 | 0.951 |
| Smoking |  |  |  |  |  |
| No | 58(55.2%) | 47(44.8%) | Ref |  |  |
| Yes | 11(52.4%) | 10(47.6%) | 1.122 | 0.439-2.868 | 0.810 |
| Alcohol intake |  |  |  |  |  |
| No | 44(58.7%) | 31(41.3%) | Ref |  |  |
| Yes | 25(49.0%) | 26(51.0%) | 1.476 | 0.721-3.020 | 0.286 |
| Use of physical exercises | | |  |  |  |
| No | 67(56.3%) | 52(43.7%) | Ref |  |  |
| Yes | **2(28.6%)** | **5(71.4%)** | **3.221** | **0.601-17.272** | **0.172** |
| Type of house | |  |  |  |  |
| Permanent | 31(62.0%) | 19(38.0%) | Ref |  |  |
| Semi-Permanent | **38(50.0%)** | **38(50.0%)** | **1.632** | **0.789-3.375** | **0.187** |
| Source of water for drinking | | |  |  |  |
| Tap water | 33(61.1%) | 21(38.9%) | Ref |  |  |
| Borehole | 32(50.8%) | 31(49.2%) | 1.522 | 0.728-3.181 | 0.264 |
| Groundwater | 4(44.4%) | 5(55.6%) | 1.964 | 0.473-8.160 | 0.353 |
| Boiling water for drinking | | |  |  |  |
| No | 50(53.8%) | 43(46.2%) | Ref |  |  |
| Yes | 19(57.6%) | 14(42.4%) | 0.857 | 0.384-1.910 | 0.706 |
| Hand washing before eating |  |  |  |  |  |
| No | 3(75.0%) | 1(25.0%) | Ref |  |  |
| Yes | 66(54.1%) | 56(45.9%) | 2.545 | 0.258-25.162 | 0.424 |
| Hand washing after the toilet | | |  |  |  |
| No | 48(53.3%) | 42(46.7%) | Ref |  |  |
| Yes | 21(58.3%) | 15(41.7%) | 0.816 | 0.374-1.783 | 0.611 |
| Number of meals per day | | | |  |  |
| < 3.0 | 19(46.3%) | 22(53.7%) | Ref |  |  |
| 3.1+ | **50(58.8%)** | **35(41.2%)** | **0.605** | **0.285-1.281** | **0.189** |

*cOR=Crude odds ratio, CI= Confidence interval, Ref= Reference category, N/A= Not applicable since the category did not register one of the outcomes.*
